# Comparison of Lens Response for Sinusoidal and Square-Wave Targets at Several Focal Positions

**DOI:** 10.6028/jres.065A.048

**Published:** 1961-12-01

**Authors:** Sayeda H. Emara

## Abstract

A study has been made of the sine-wave and square-wave responses of a lens at two apertures and several focal positions, both on- and off-axis. Two focal positions, one of which gives the best definition and the other the highest contrast for coarse patterns, were located precisely. At these focal positions and at several other arbitrarily chosen positions the sine-wave and square-wave responses were measured. Because of the scattering character of the photographically-made target objects, a special technique has been employed for calibrating the targets to obtain their contrast as seen by the lens under test.

The results show that for large apertures of the lens there is some frequency at which the response (either sine or square) is the same for the two focal positions (curves cross). This phenomenon has been further studied by computing the square-wave response of the lens from its sine-wave response; and it was found that there is close agreement between the computed and experimentally determined responses.

## 1. Introduction

During the past few years the National Bureau of Standards and a number of other laboratories have been engaged in a research program with the aim of developing a more objective method of assessing the image qualities of a lens than those methods currently in use.

Among numerous procedures, the one based upon Fourier analysis of the object and the image into sinusoidal components is of great theoretical interest. The striking feature of the Fourier approach is the simplicity with which sinusoidally varying luminance patterns are imaged. Diffraction and aberrations have no effect whatsoever on the sinusoidal shape of the image of such a wave, nor on its orientation. In other words, the image of a sinusoidal pattern is also sinusoidal; only the amplitude (or modulation factor) is changed by the imaging process. Thus the performance of an image-forming system may be described by a graph or table that gives the degradation of contrast as a function of spatial frequency for sinusoidal objects. Two such graphs or tables for sinusoidal objects at right angles to each other completely describe the image-forming characteristics at one point in the field.

The easiest method for determining these characteristics makes use of the multiple line object and has been used by several workers [1, 2, 3]. For this method the image of the lines is scanned by a narrow slit to measure the distribution of flux in that image. Recently an improved technique was developed at the National Bureau of Standards which scanned the aerial image directly with a slit and photo-multiplier tube [4]. The resultant variations in illuminance in the image were recorded on the moving chart recorder.

One might suppose that the proper focal plane at which the measurements should be performed would be easily determined as the one at which the response (or transfer function) was maximum. Unfortunately patterns of different frequency give different focuses for their respective maximum responses [5]. Consequently, the selection of the proper focus must be judged by additional criteria. Many investigators have reported the existence of different best focuses, depending upon the criteria. There is a focus that gives the best resolution of fine detail and, presumably, as a consequence, the sharpest reproduction of edges. There is another focus that gives best contrast of coarse detail. If only coarse detail is important, as in 530-line television pictures, this focus may be more nearly optimum than the other. In a previous work a comparison was made between the focal position for highest contrast and the position for best definition, using a coarse parellel line target [6]. Examination of photographs of the pattern indicated a maximum sharpness of edges at one focal position while the maximum amplitude of the traces gave a different position of highest contrast, with a separation of several tenths of a millimeter between the two positions for the particular lens tested. Although the results showed this discrepancy between the focal positions of highest contrast and best definition in a spatial frequency area somewhat removed from the limit of resolution, near this limit the two criteria were satisfied at the same position.

The conclusion arrived at for the location of the position of best definition were based entirely upon visual judgment. Also, all observations were carried out using only one coarse pattern and one position, off-axis. Therefore, it was felt that further study of the problem, using more precise measurements, should be made. Thus it is the aim of this paper to present accurate procedures to locate the focuses of best definition and highest contrast and then to measure the sine-wave response and the square-wave response over a wide range of frequencies and apertures for a given lens in the hope of finding a satisfactory explanation of this phenomenon.

Although the work was done with only one lens, a Zeiss Biotar, *f*/2, with a focal length of 58 mm, the results are representative of the performance of practically all high grade photographic lenses.

## 2. Arrangement of Apparatus

**Figure 1:**
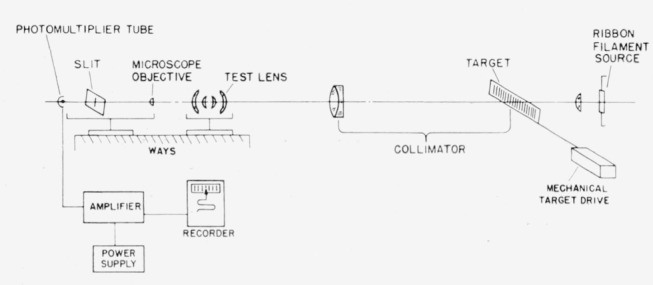
Schematic diagram of the apparatus used.

The apparatus was as shown in figure 1. The source was a ribbon filament tungsten lamp focused by a condensing lens on the target located at the focus of the collimator. Thus illumination in this case fulfills the conditions for noncoherence [7]. The lens under test was located on a nodal slide in the collimated beam. The nodal slide and pickup unit were mounted on separate saddles riding on a length of ways alined with the optical axis established by the collimator.

Immediately behind the test lens was the pickup unit consisting of a microscope, slit, and photo tube. In operation the object was moved in the focal plane of the collimator so that the image moved across the slit.

## 3. Description of Targets

The two targets used in this investigation were transparencies on glass plates, with a transmittance variation forming a square wave for one and a sine wave for the second. There was no variation in transmittance in the orthogonal direction. The square-wave target consisted of 25 patterns, each of which had three dark lines, with lines and spaces of equal width. The spatial frequencies in the image of the patterns ranged from the coarsest, of 6.12 lines per millimeter, to the finest, of 97.6 lines per millimeter. The sine-wave target had 23 patterns producing spatial frequencies in the image of 3 lines per millimeter for the coarsest pattern and approximately 500 lines per millimeter for the finest pattern.

The target under investigation was mounted on a carriage and moved through the light beam in a plane parallel to the image plane along a line at right angles to the lines on the target. The scanning speed was constant for a given pattern but for coarse patterns it was faster than for fine patterns. An effort was made to maintain a constant scanning rate in terms of lines per minute for the entire target by changing target speed with gears.

## 4. Calibration of Targets

Both targets were calibrated using a Leeds and Northrup microdensitometer. The numerical apertures of the microscope and condenser lenses of the microdensitometer were approximately 0.35, whereas those of the collimator and lens being tested were very small, always less than 0.008. This difference of geometry between the system in which the targets were calibrated and that in which they were used resulted in an error. This error was discovered when the measured sine wave responses of the lens were apparently greater than one for some of the coarse patterns. This cannot, of course, be correct. The discrepancy was attributed to the fact that the contrast of photographic materials appears greater with small apertures than with large because of their scattering nature.

It is not possible to eliminate this error by performing the calibration with the aperture of the microdensitometer reduced so as to match the conditions of use. Such a procedure would so reduce the resolving power of the microdensitometer as to render the calibration useless at the higher frequencies. Consequently an indirect calibration process was adopted.

**Figure 2:**
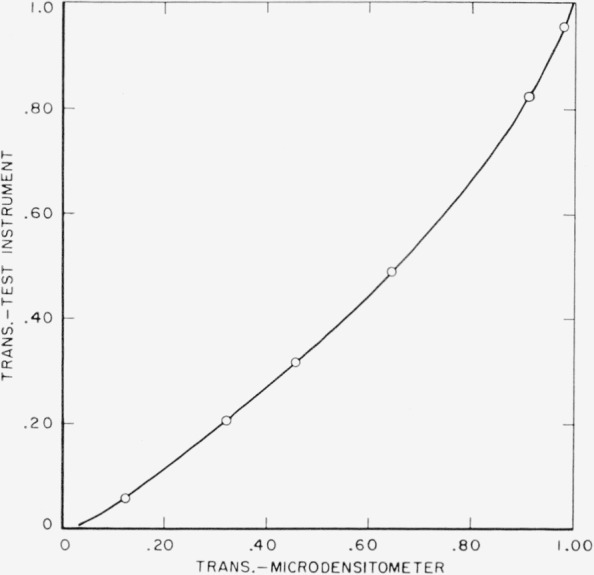
Calibration curve for transmittance measured by the test instrument.

For this calibration a photographic plate was exposed to give a series of fairly broad strips, about 1 cm in width, varying in transmittance by small increments over the entire density scale of the plate. The type of plate was chosen in order to have similar scattering characteristics to that on which the target objects were made. The transmittances of the strips on this plate were measured on the microdensitometer; they were also measured on the test instrument at a numerical aperture of approximately 0.008. The widths of the strips were sufficient that the accuracy of these measurements was not reduced by the low resolving power of the system. The values obtained are exactly the same as if the lens system were free from aberrations and diffraction. A calibration curve was made by plotting transmittance values by test instrument as a function of transmittance by microdensitometer using the data obtained with the step density plate. This is shown in figure 2.

Obviously the end points, zero and unity, are the same on both instruments, but in between there is considerable difference. Correction of target modulations by means of this calibration eliminated responses greater than one and led to satisfactory agreement between measured and computed square-wave responses.

## 5. Procedure

**Figure 3:**
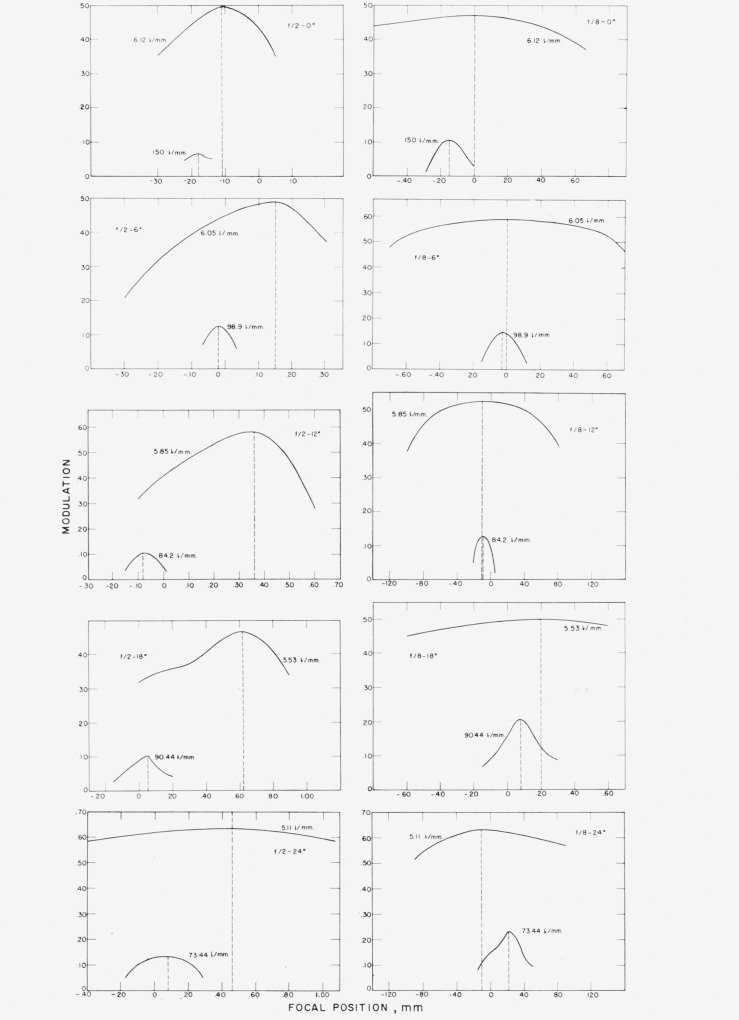
Curves used for obtaining the focal positions for best definition and highest contrast.

The initial requirement of each run was to determine the focal positions of best definition and highest contrast for the selected *f*-stops. Runs were made at lens stop openings of *f*/2 and *f*/8. The position of best definition was determined by using a pattern near the limit of resolution but still giving enough modulation, compared to the noise level, to be measured with moderate accuracy. Several scans were made of the same target pattern with small change in focal position between scans to determine the position yielding the largest signal or modulation. Modulation of the image was calculated using the formula

$$M_{i}=\frac{T_{\max}-T_{\min}}{T_{\max}+T_{\min}},$$

where *M_i_* is the image modulation produced by the lens under test, *T*_max_ the illuminance of a space, and *T*_min_ the illuminance of the adjoining line. A plot of image modulation against focal position was drawn. The maximum modulation of this curve determined the position for best definition. The procedure was repeated using a coarse pattern on the square-wave target and a similar curve was drawn to indicate the position of highest contrast. There was little difficulty in finding these focal positions of the lens on axis but at off-axis positions the increased depth of focus of the lens gave flat-top curves, especially for the highest contrast positions, as can be seen in figure 3.

Several runs were made at other apertures, *f*/2.8, *f*/4 and *f*/5–6, where it was found that there was practically no difference between the positions of best definition and best contrast. This is an interesting phenomenon itself, which is worthy of further investigation; but, as we were interested in cases where these two focuses were distinct, no further results are reported for these apertures.

After the correct focal positions had been obtained, the response was determined for several patterns on each target and a plot was made of response as a function of frequency for each focal position. This was accomplished by moving the target through the light beam parallel to the image plane and tracing the resultant image illuminances on the recorder chart. A pattern consisted of a set of lines and spaces of constant spatial frequency. Thus, the target pattern produced a spatial variation in luminance consisting of a sinusoidal component superimposed on a constant component. The ratio of the amplitude of the sinusoidal component to the magnitude of the constant component is the target modulation. An imperfect image of this pattern was formed by the lens under test. This image was further projected onto the plane of the receiver slit with a good low-power microscope objective. Thus the distribution of illuminance in the image was measured.

When the target is imaged by the lens under test, the modulation is reduced, the degree of reduction depending on the quality of the lens. The ratio of the modulation in the image to that in the object is called the response. This factor generally diminishes as the spatial frequency of the pattern increases, the lens acting as a low-pass filter on spatial frequencies. Thus if *M_t_* denotes the modulation of the target and *M_i_* that of the image produced by the lens under test, then the ratio of the two is called response,

$$\Phi =\frac{M_{i}}{M_{t}}.$$

The use of the modulation automatically compensates for any factor which changes the signal output of the testing device proportionally such as the transmission factor of the lens, change in brightness of the source, change in gain of the detector amplifier, etc. It must be remembered that the complete response function is actually complex, involving both an amplitude and a phase factor, of which Φ is the amplitude factor. However, for measurements made on axis there is no phase shift involved. In most cases, for evaluation purposes, the phase shift factors can be neglected.

The square-wave response of the lens was computed from its sine-wave response, employing Fourier analysis. A general object was considered to be the summation of a set of sinusoidal waves spatially distributed in the object plane, these component waves differing from each other in amplitude, frequency, phase, and direction. The image consisted of the summation of the images of these component waves.

The square-wave test object can be analyzed into its component harmonics, each of which is attenuated by the Φ corresponding to its frequency, and the square-wave response is obtained by adding these attenuated harmonics [8]. For the response curve we are only interested in the peak-to-peak values which are obtained from the values at the centers of the lines and spaces. The square-wave response curve on axis is then given by:

$$\Psi (\omega )=\frac{4}{\pi }\left[\Phi (\omega )-\frac{1}{3}\Phi (3\omega )+\frac{1}{5}\Phi (5\omega )-\ldots \right].$$ (1)

It should be noticed that there will be only a finite number of terms in the sum because there is a limiting value of *ω* beyond which Φ is zero for all larger values of *ω*. This limit exists because of the finite dimensions of the aperture of the system the larger the aperture, the higher the limiting frequency.

**Figure 4:**
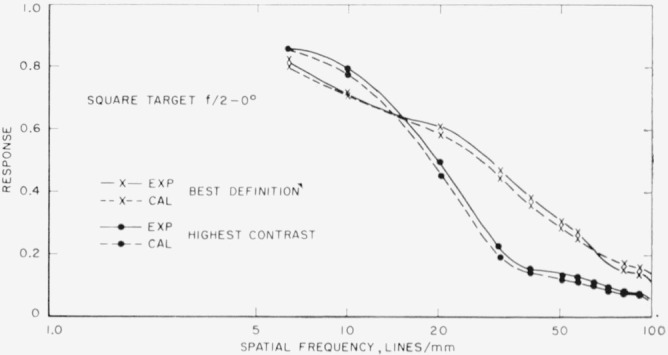
Best definition and highest contrast curves for the square-wave target at *f/2* on axis. The two solid curves are those obtained by experiment while the two dotted curves are for values calculated from the sine-wave response using Fourier analysis.

Figure 4 shows square-wave target curves or axis using a lens stop of *f*/2. The two solid curves are the experimental curves plotted from data taken in the focal positions of best definition and highest contrast, while the two dotted curves are obtained from values calculated from eq (1). The agreement between the experimental and the calculated curves is very good.

It is to be emphasized that comparison of measured square-wave responses with computed values was entirely unsatisfactory until averages of several runs were used for both the square-wave and sine-wave responses used in the computation. Accordingly, care has been taken to have an average of several runs for each curve shown in this investigation. To convey an idea of the magnitudes of the uncertainties of these results, tables 1 and 2 show the average responses and associated probable errors for two different focuses, best definition and highest contrast, respectively, for the square wave target. These tables represent the results of five runs each.

## 6. Results

**Figure 5:**
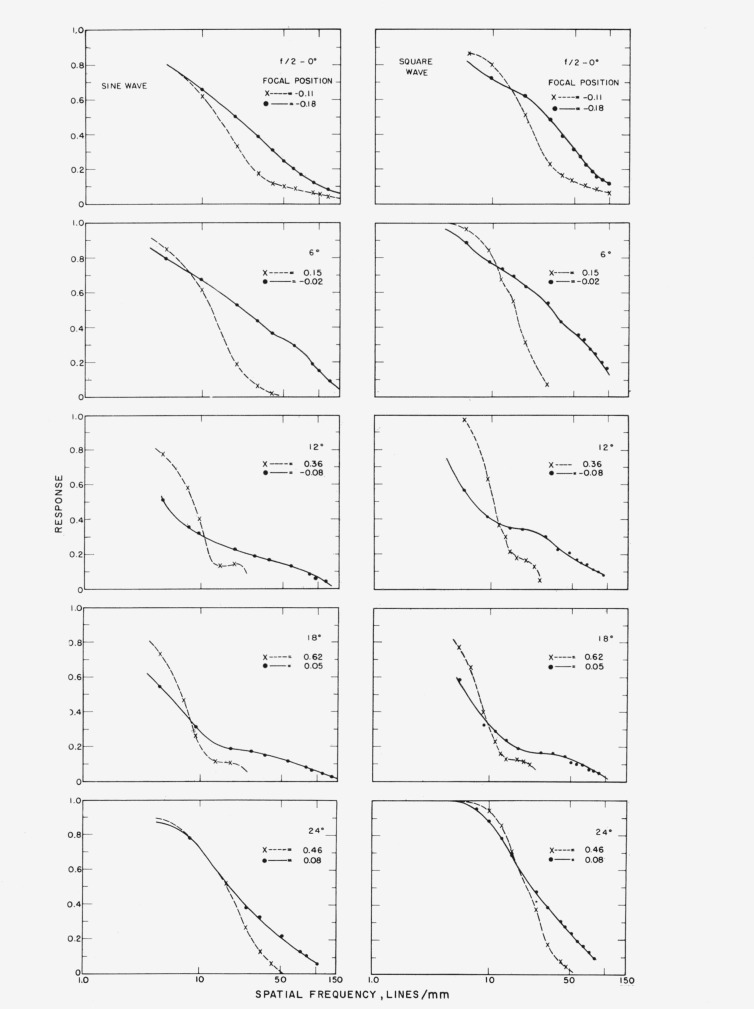
Responses for square-wave and sine-wave targets at *f/2* on axis and for four off-axis positions, 6°, 12°, 18° and 24°.

Figure 5 illustrates the square- and sine-wave response curves at an *f*/2 stop opening on axis and at off-axis positions of 6°, 12°, 18°, and 24°. The curves for the square-wave show an interesting phenomenon. On axis the best-definition and highest-contrast curves intersect at a spatial frequency of 14.5 lines per millimeter with a response of 0.66. At 6° off-axis best-definition and highest- contrast curves intersect at the spatial frequency of 11 lines per millimeter, where the response is 0.65. At 12° these curves intersect at a point where the frequency is 11.5 lines per millimeter at a lower response of 0.39. At 18° the frequency at which they intersect reaches a value of approximately 9.6 lines per millimeter at a response as low as 0.34. At 24° the two curves intersect at a spatial frequency of 17 lines per millimeter at a response of 0.65. The curves for the lens at the position of highest contrast show that the finest pattern resolved decreases in frequency at off-axis positions and reaches a minimum of 23 lines per millimeter at 18° but increases again at 24° reaching a value of 46 lines per millimeter, while the best-definition curves show little change.

The sine wave shows characteristics similar to those for the square wave. On-axis the behavior is as expected; but at 6° off-axis the highest contrast and best definition curves intersect at a frequency of 7.8 lines per millimeter, where the response is 0.71. At 12° the two curves intersect at a spatial frequency of 10.8 lines per millimeter with a decreased response of 0.31. At 18° off-axis the spread between the best definition and highest contrast decreases with coincidence at a spatial frequency of 8.2 lines per millimeter and a response of 0.36. As the lens is rotated further about its vertical axis until 24° off-axis is reached the best definition and highest contrast curves intersect at a spatial frequency of 8.8 lines per millimeter with a response of 0.77. It is of interest to note that at this frequency the response has again reached a higher value, closer to that obtained on axis. The finest pattern resolved for the highest contrast curves decreases in frequency for increasing off-axis angles, reaching a minimum of 18 lines per millimeter at 18° and then increasing to a value of 42 lines per millimeter at 24°.

This suggests that for both targets there is a serious effect from aberrations reaching a maximum near 12° off-axis and then decreasing as off-axis positions approach 24°. This obviously is a consequence of the zonal nature of the corrections in photographic lenses. In this case the observations appear to be most nearly corrected on axis and at about 24° off-axis with some deterioration of image quality in between.

**Figure 6:**
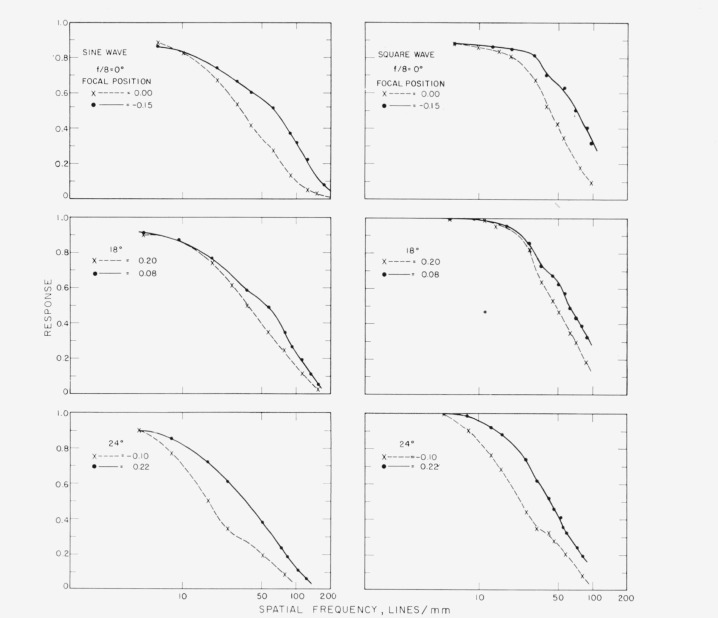
Responses for square-wave and sine-wave targets at *f/8* on axis and for two off-axis positions, 18° and 24°.

Figure 6 shows a set of curves for both targets using *f/8* stop of the lens on axis and at 18° and 24° off-axis positions. It is clearly seen that the results obtained with the two targets are similar. The best definition and highest contrast curves differ considerably on axis and at 24° off-axis while the difference at 18° is smaller.

Curves at 6° and 12° with an *f*/8 stop opening are omitted because at these off-axis positions the focal positions for best definition and highest contrast were very close and no appreciable difference in response could be observed on these curves.

**Figure 7:**
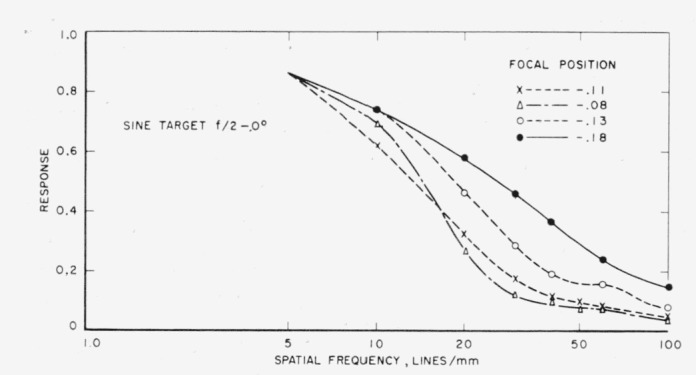
Sine-wave response curves for four different focal positions on-axis.

**Figure 8:**
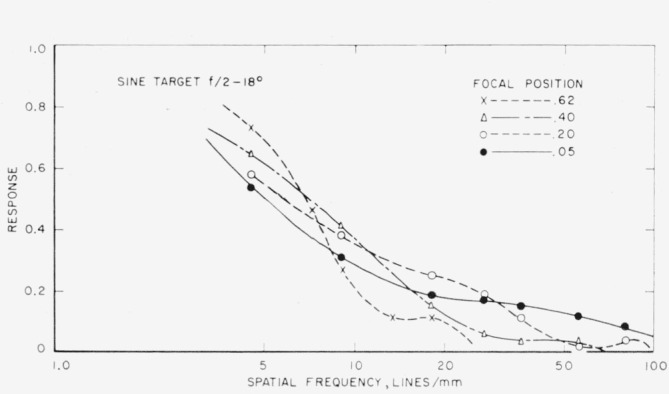
Sine-wave response curves for four different focal positions at 18° off-axis.

Further runs were made, both on- and off-axis, using the *f*/2 lens stop and four different focal positions, two of which were the positions of highest contrast and best definition. Two examples are given illustrating the effect of changing the focal positions. Figure 7 shows curves for the sine-wave target at four different focal positions on axis of the lens. The best focal position is that of best definition. Figure 8 illustrates another set of curves at 18° off-axis. Here again it is clearly seen that in the spatial frequency range of 5 to 6.8 lines per millimeter the highest contrast position (0.62) gives the highest response while in the range of 6.8 to 12 lines per millimeter the position next to highest contrast (0.40) is best. In the area 12 to 29 lines per millimeter the next focal position (0.20) is best and, finally, from 29 lines per millimeter up the position of greatest definition (0.05) is best.

## 7. Conclusions

There are a number of conclusions which can be drawn:

1. The square-wave response can be predicted reasonably well if the sine-wave response is known. It is concluded that for a large aperture of the lens there is a certain spatial frequency at which the best definition and highest contrast curves coincide. Its value differs slightly for off-axis positions.
2. The response for both targets decreases as spatial frequency increases.
3. There is a serious effect caused by lens aberrations at off-axis positions; reaching a maximum for this lens close to 12° and decreasing again at 24°.
4. Displacement of focal positions at large apertures shows that the position of best definition may be inferior for some purposes.
5. Curves drawn for locating the focuses of best definition, and especially those for locating focuses of highest contrast become very flat topped for small apertures. Consequently these two focuses become indistinguishable. This is, of course, a manifestation of the increasing of depth of focus.

## Figures and Tables

**Table 1:** Responses to square waves at the focus of best definition with associated probable errors; measured on axis at f/2.

Lines/mm	6.12	9.7	19.4	30.7	38.7	49.2	55.2	62.4	69.8	78.5	88.4	97.5
Average response	0.820	0.720	0.626	0.492	0.384	0.310	0.272	0.220	0.177	0.151	0.142	0.111
Probably error	±0.025	±0.027	±0.021	±0.018	±0.018	±0.012	±0.012	±0.008	±0.008	±0.007	±0.008	±0.006

**Table 2:** Responses to square waves at the focus of highest contrast with associated probable errors; measured on axis at f/2.

Lines/mm	6.12	9.7	19.4	30.7	38.7	49.2	55.2	62.4	69.8	78.5	88.4	97.5
Average response	0.860	0.800	0.514	0.228	0.158	0.138	0.124	0.114	0.097	0.084	0.079	0.060
Probable error	±0.027	±0.026	±0.035	±0.015	±0.008	±0.014	±0.007	±0.006	±0.007	±0.007	±0.005	±0.002
